# Comparison of serum and oral fluid antibody responses after vaccination with a modified live (MLV) porcine reproductive and respiratory syndrome virus (PPRSV) vaccine in PRRS endemic farms

**DOI:** 10.1007/s11250-015-0868-6

**Published:** 2015-06-13

**Authors:** Ah Meng Kuiek, Peck Toung Ooi, Chiun Khang Yong, Chi Foon Ng

**Affiliations:** Department of Veterinary Clinical Studies, Faculty of Veterinary Medicine, Universiti Putra Malaysia, 43400 Serdang, Selangor Malaysia; Boehringer Ingelheim (Malaysia) Sdn Bhd, Kuala Lumpur, Malaysia; IDEXX Laboratories (Malaysia), Kajang, Malaysia

**Keywords:** Porcine reproductive and respiratory syndrome (PRRS), Oral fluid, IDEXX PRRS oral fluid test kit, IDEXX PRRS X3 test kit

## Abstract

Porcine reproductive and respiratory syndrome (PRRS) is a disease that is both highly contagious and of great economic importance in Malaysia. Therefore, reliable and improved diagnostic methods are needed to facilitate disease surveillance. This study compared PRRSV antibody responses in oral fluid versus serum samples following PRRS modified live (MLV) vaccination using commercial antibody ELISA kits (IDEXX Laboratories, Inc.). The study involved two pig farms located in Perak and Selangor, Malaysia. Both farms were vaccinated with PRRS MLV 1 month prior to sample collection. Thirty-five animals were used as subjects in each farm. These 35 animals were divided into 7 different categories: gilts, young sows, old sows, and four weaner groups. Oral fluid and serum samples were collected from these animals individually. In addition, pen oral fluid samples were collected from weaner groups. The oral fluid and serum samples were tested with IDEXX PRRS Oral Fluid Antibody Test Kit and IDEXX PRRS X3 Antibody Test Kit, respectively. The results were based on sample to positive ratio (S/P ratio of the samples). Results revealed a significant and positive correlation between serum and oral fluid samples for both farm A (*p* = 0.0001, *r* = 0.681) and farm B (*p* = 0.0001, *r* = 0.601). In general, oral fluids provided higher S/P results than serum, but the patterns of response were highly similar, especially for the sow groups. Thus, the use of oral fluids in endemic farms is effective and economical, particularly for large herds. In conclusion, the authors strongly recommend the use of oral fluids for PRRS monitoring in endemic farms.

## Introduction

Porcine reproductive and respiratory syndrome (PRRS) is one of the major threats to the pig industry and can cause great economic loss due to reproduction failure in sows and preweaning mortality of up to 60 %. This will also cause significant economic loss to the pig farmers as they are not able to increase the production of the farm. Based on a recent survey, it is estimated that losses due to PRRS problems in the USA are as high as $668.58 million annually (Zimmerman et al. 2012). PRRS continues to be a major economically important disease in swine and has been demonstrated in Asian countries such as China, Philippines, Vietnam, Cambodia, Laos, Thailand, and Myanmar (Na Ayudhya et al. 2012). It is also believed that PRRS in Vietnam spreads from China because the isolates from both countries show 99 % identical genomes (Zhang and Kono 2012). In Malaysia, a recent seroprevalence study indicated that 89.2 % of the sera tested were seropositive against PRRS virus (Vania and Ooi 2012). This indicates the importance of proper monitoring of PRRS in Malaysian pig farms.

In general, a tentative diagnosis of PRRS can be made base on clinical signs, such as reproduction problems in breeding stocks and respiratory disease. However, since clinical signs of PRRSV are not consistent and the virus does not cause specific lesions, differential tests are needed to achieve a definitive diagnosis. Based on the clinical signs of reproduction and respiratory problems, the differential diagnosis includes porcine parvovirus infection (PPV), porcine circovirus type-2 infection (PCV2), and classical swine fever (CSF). Hence, when the clinical signs and postmortem findings are suggestive of PRRS, detection of viral antigens, viral genomic material, or isolation of virus from clinical specimens is necessary to confirm the diagnosis. Besides that, rising serum antibodies against PRRSV can also be used to support the diagnosis, provided that the time frame is compatible with the clinical episode.

There are several ways to monitor PRRS status, including detection of serum antibodies using commercial PRRS enzyme-linked immunosorbent assays (ELISAs), reverse-transcriptase polymerase chain reaction (RT-PCR) assays, frozen tissue section fluorescent antibody (FA) assay, and immunohistochemistry (IHC). Detection of pathogen-specific antibodies by ELISA is one of the most common methods for detecting PRRSV infection. The ELISA format makes it possible to test and analyze a large number of serum samples, which can reduce cost and labor involved. However, blood sampling in pigs is laborious, time consuming, and requires restraining of individual pigs. Therefore, a novel method of detecting PRRS in pigs is needed.

Oral fluid is collected by placing an absorptive device, e.g., cotton rope, in the mouth. The use of cotton rope to collect oral fluid samples from pigs has been done successfully under experimental and field conditions (Prickett et al. 2008). Oral fluid samples contain both serum transudate and saliva from the animal. Serum transudate contains a variety of pathogens and antibodies from the animal. Hence, it is possible to use oral fluids as diagnostic samples for epidemiological studies. In livestock, oral fluid has not been widely used for testing, but the veterinary literature does report the presence of antibodies, pathogens, and acute phase proteins in oral fluid. For example, in swine, infectious agents, cortisol, acute phase proteins, and progesterone have all been detected in oral fluid samples in both experimental and field conditions (Kittawornrat et al. 2012). Thus, the purpose of the present study was to compare the oral fluid and serology method in PRRS modified live (MLV) vaccinated in PRRS endemic farms.

## Materials and methods

### Animals

This study was conducted at two commercial pig farms located in the central part of Malaysia. Both farms practiced intensive, farrow-to-finish, open-house systems. Pigs in both farms were vaccinated with PRRS MLV vaccine 1 month prior to sampling. Both farms practiced sow PRRS MLV mass vaccination and piglet vaccination at day 14. Thirty-five pigs of different age groups and breeding stages were randomly selected as subjects from each farm. In general, the pigs from each farm were divided into seven different groups based on their ages and breeding cycles: (1) sows ≥six parities, (2) sows two to five parities, (3) gilts, (4) 10-week-old pigs, (5) 15-week-old pigs, (6) 20-week-old pigs, and (7) 25-week-old pigs.

### Sampling

Oral fluid samples were collected using three-strand twisted undyed cotton rope. Both individual and pen oral fluid samples were collected. For individual oral fluid samples, pigs were identified based on their age group and record. After subjects were identified, the cotton rope was offered and the pigs were allowed to chew for 20 min. Individual oral fluid samples were collected from all seven groups, with five subjects from each category selected for collection. For individual oral fluid sampling, other pigs were prevented from chewing the same rope to avoid cross contamination. For pen oral fluid sampling, the ropes were hung inside the pen for 20 to 30 min. Pen oral fluid samples were collected from groups 4 to 7, only. Pen oral fluid samples were collected three times as replicates. Oral fluid collection was done by inserting the wet end of the rope into a clean plastic bag. The rope was squeezed slowly to remove the fluid into the collection tubes. Blood samples were collected from pigs via the jugular vein. Serum was then extracted from the collected blood and stored under −20 °C for further processing.

### Serological tests

Serum samples were tested using the IDEXX PRRS X3 Antibody Test Kit, and oral fluid samples were tested using the IDEXX PRRS Oral Fluid Antibody Test Kit. Both test kits use the indirect ELISA format. Test results were expressed by calculating the sample-to-positive control (S/P) ratio for each sample using commercial software (IDEXX XCheck^®^ software).

### Statistical analysis

S/P ratios for both oral fluid and serum samples were analyzed with SPSS version 20. Pearson’s product-moment correlation test was used to determine the correlation between oral fluid and serum samples. For pen oral fluid samples, paired samples *t* test was used to identify whether there was a difference between individual and pen oral fluid samples for the same age group.

## Results

The pig population in each farm was divided into seven categories: (1) sows ≥six parities, (2) sows two to five parities, (3) gilts, (4) 10-week-old pigs, (5) 15-week-old pigs, (6) 20-week-old pigs, and (7) 25-week-old pigs. Both blood and oral fluid samples were taken from all individual pigs from all seven categories. In addition, pen oral fluid samples were collected from categories 4, 5, 6, and 7.

**Fig. 1:**
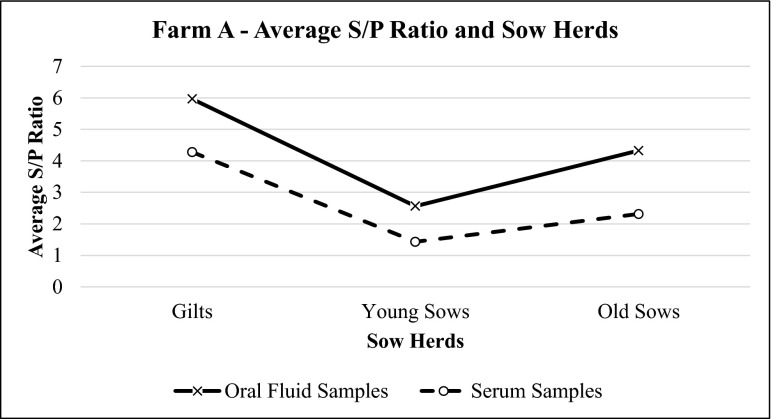
Average S/P ratio for oral fluid and serum samples of farm A sow herds based on IDEXX PRRS OF Ab test and IDEXX PRRS X3 Ab test results, respectively. The values of S/P ratio show the same trend for both oral fluid and serum samples

**Fig. 2:**
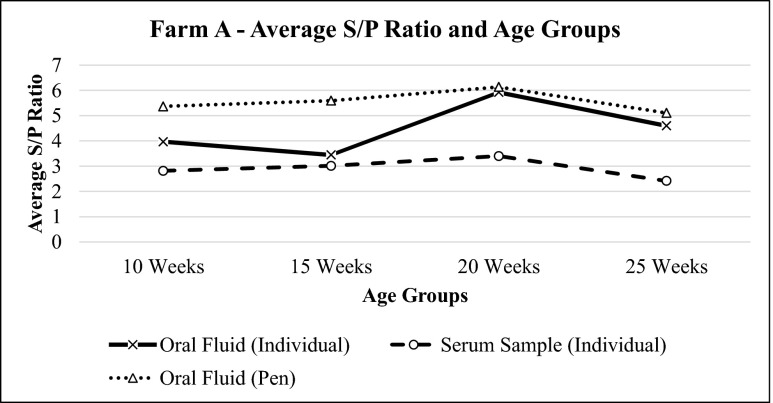
Average S/P ratio for oral fluid and serum samples of farm A porkers based on IDEXX PRRS OF Ab test and IDEXX PRRS X3 Ab test results, respectively. The values of S/P ratio show similar trend for both types of samples of all age groups in general except for porkers at 15 weeks which shows that individual oral fluid samples have sharp increase trend compared to serum samples

Results showed that for farm A, oral fluid had higher S/P ratios than serum samples (Fig. 1). Similar results were observed for the weaner and grower group (Fig. 2). Literature suggested that S/P values which are considered normal for serum (0.5 to 1.5) would have higher values for oral fluid (3.0 to 6.0) (IDEXX 2013).

**Fig. 3:**
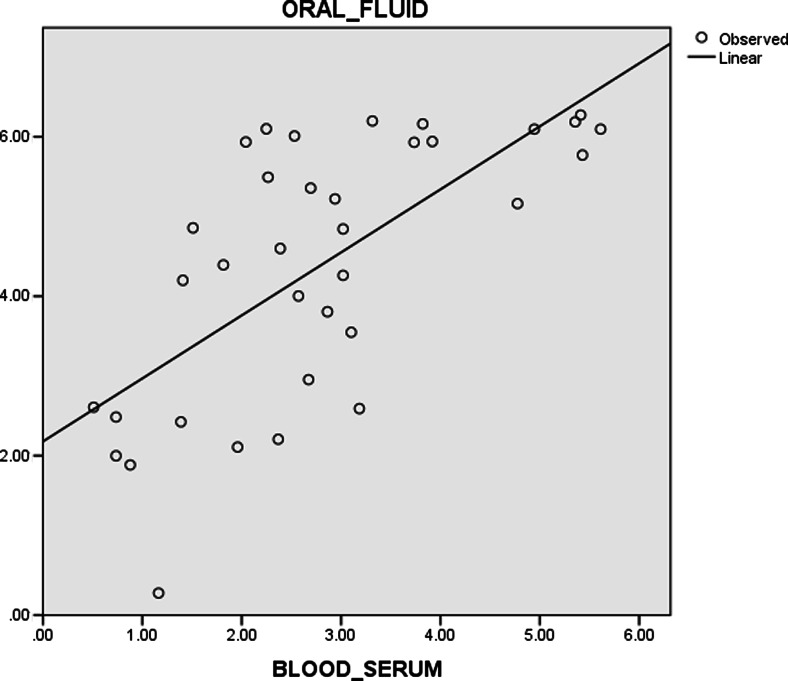
Correlation between S/P ratios for oral fluid and serum samples from individual subjects in farm A as a summary statistic (Pearson’s correlation coefficient, *r* = 0.681)

Both oral fluid and serology samples show positive, significant, and strong correlation (*p* = 0.0001, *r* = 0.681) using Pearson’s correlation test (Fig. 3). This strong correlation coefficient was further supported by a coefficient of determination (*r*^2^) of 0.464. This means that about 46.4 % of the total variation in S/P values of oral fluid samples can be explained by variation in S/P values of serum samples.

**Fig. 4:**
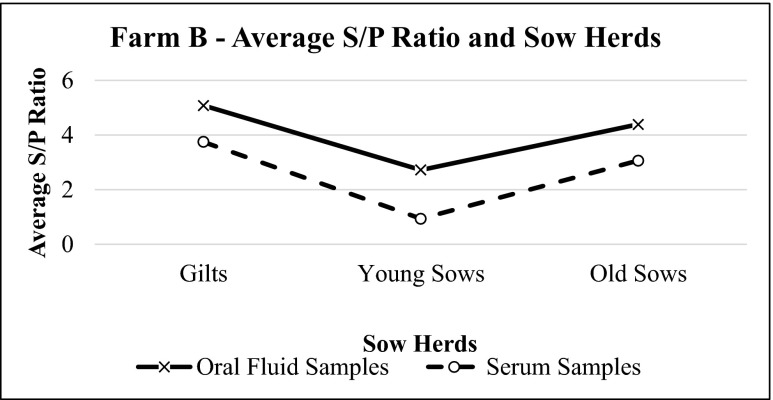
Average S/P ratio for oral fluid and serum samples of farm B sow herds based on IDEXX PRRS OF Ab test and IDEXX PRRS X3 Ab test results, respectively. The values of S/P ratio show the same trend for both oral fluid and serum samples

**Fig. 5:**
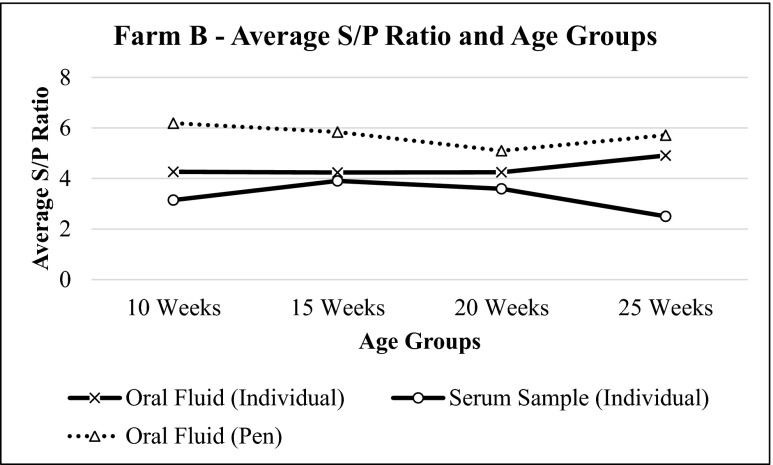
Average S/P ratio for oral fluid and serum samples of farm A porkers based on IDEXX PRRS OF Ab test and IDEXX PRRS X3 Ab test results respectively. The values of S/P ratio show inconsistent trend regardless oral fluid or serum samples

**Fig. 6:**
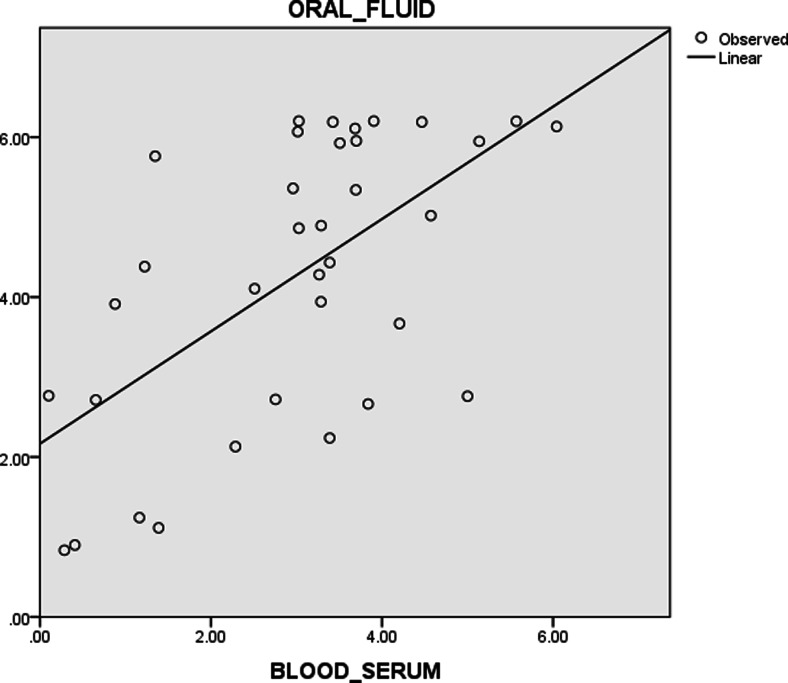
Correlation between S/P ratios for oral fluid and serum samples from individual subjects in farm B as a summary statistic (Pearson’s correlation coefficient, *r* = 0.601)

The results for farm B also showed similar pattern for both oral fluid and serum samples. Oral fluid samples consistently had higher S/P values, when compared to serum samples. The trend of S/P ratios for sow herds in this farm was similar to farm A (Fig. 4), while the trend for porkers varied (Fig. 5). Pearson’s product-moment correlation test results showed positive, significant, and strong correlation between these two sample types (*p* = 0.0001, *r* = 0.601) with a coefficient of determination (*r*^2^) equal to 0.369 (Fig. 6).

**Fig. 7:**
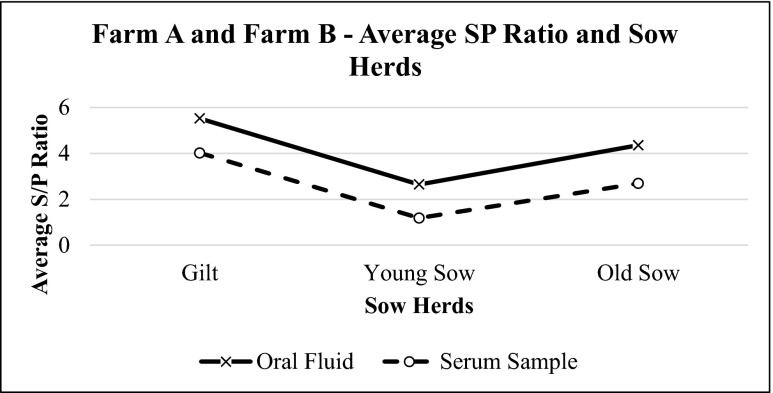
Average S/P ratio for oral fluid and serum samples of farm A and farm B sow herds based on IDEXX PRRS OF Ab test and IDEXX PRRS X3 Ab test results, respectively. The values of S/P ratio show similar pattern for both samples

**Fig. 8:**
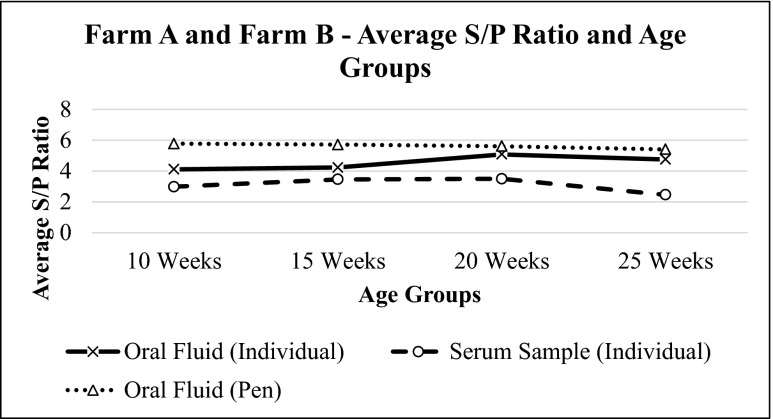
Average S/P ratio for oral fluid and serum samples of farm A and farm B at different age groups based on IDEXX PRRS OF Ab test and IDEXX PRRS X3 Ab test results, respectively. The values of average S/P ratio of individual oral fluid and serum samples show similar pattern at different age groups. Meanwhile, average S/P ratios for pen oral fluid samples do not deviate much at different age groups

**Fig. 9:**
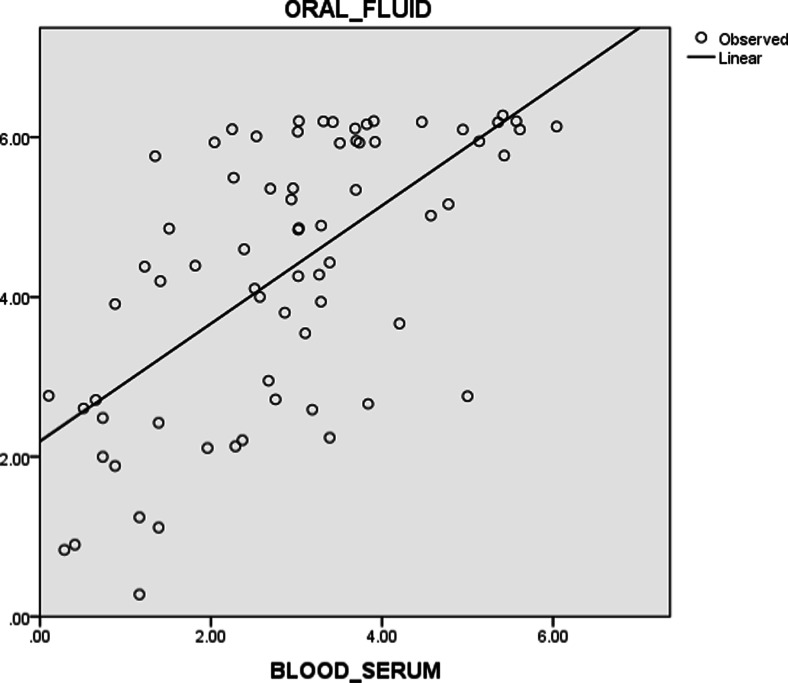
Correlation between S/P ratios for oral fluid and serum samples from individual subjects in both farms as a summary statistic (Pearson’s correlation coefficient, *r* = 0.638)

Oral fluid and serum sample results from both farms were also evaluated together. Oral fluids and serum samples showed similar patterns at different age groups in sows (Figs. 7 and 8) and were statistically correlated with each other (*p* = 0.0001, *r* = 0.638) (Fig. 9).

Pen oral fluids for the following age groups were also collected: 10 weeks old, 15 weeks old, 20 weeks old, and 25 weeks old. Three replicates were taken and tested with PRRS OF Ab Test Kit. These results were compared with individual samples.

Statistical analysis of farm A found no difference between the results of pen and individual oral fluid samples for the same age group (*p* = 0.094). This means that there is no difference between these two samples at 95 % confidence interval. On the other hand, analysis of farm B determined that the results of pen and individual samples of same age group were the same, with the exception of week 20 (*p* = 0.05) (Table 1).

**Table 1:** Summarized mean S/P ratio for farm A and farm B pen and individual oral fluid samples for different age groups

S/P ratio				
Age	Farm A		Farm B	
	Pen samples (mean ± SE)	Individual samples (mean ± SE)	Pen samples (mean ± SE)	Individual samples (mean ± SE)
10 weeks	5.369 ± 0.279	3.969 ± 0.727	6.185 ± 0.022	4.263 ± 0.684
15 weeks	5.594 ± 0.186	3.445 ± 0.659	5.835 ± 0.093	4.233 ± 0.733
20 weeks	6.136 ± 0.030	5.925 ± 0.117	5.098 ± 0.174	4.244 ± 0.659
25 weeks	5.108 ± 0.312	4.600 ± 0.240	5.715 ± 0.222	4.907 ± 0.514

## Discussion

From this experiment, there are significant, positive, and strong correlations between oral fluid and serum samples for both farms A and B. This positive and significant correlation indicates that increase in S/P ratios of serum samples will also cause increase in S/P ratios of oral fluid samples. This proves that oral fluid samples can be used as PRRS monitoring tool instead of serum samples as they are closely correlated to each other. A comparison had also been done on matched samples from individual boars, and it revealed that oral fluid was equal to serum for the detection of PRRSV at DPI 7 and more likely to be more positive than serum on DPI 14 and 21 (Kittawornrat et al. 2013). Hence, this suggests that oral fluid is superior to serum over a 21-day observation period.

Besides that, from this experiment, it can be seen that sow herds have a more consistent result for both oral fluid and serology detection methods. Sow immunity status might be more stable if compared to porkers. In porkers, the immunity challenge from the field might be higher, and therefore, the immunity level may vary, which leads to different S/P value fluctuation among age groups.

The most common method for monitoring PRRSV infection in swine populations is by using IDEXX PRRS X3 Antibody Test Kit, which uses serum as specimens. This approach is laborious and time consuming and may pose danger to the workers and practitioners if the subjects are not cooperative. Typically, only a few randomly chosen animals are selected to represent the immune status of the entire farm. This approach is unable to achieve enough sampling requirements for a targeted level of disease detection because it is impossible to do blood sampling for all the animals in the farm (Kittawornrat et al. 2012).

Oral fluid is a good technique to be used as a monitoring tool for PRRS in the farm because a large number of animals can be sampled at one time. This study showed that the pattern of the oral fluid antibody response was similar to that seen in serum. Therefore, it will be a useful and reliable sample to replace the conventional serum as PRRS monitoring in the endemic farm. Besides that, oral fluid collection can also be done easily by only one person on a daily basis without posing danger to the personnel. At the same time, pigs have a natural behavior of chewing anything surrounding them, thus making it easier for the samples to be collected (Kittawornrat et al. 2012). Oral fluid collection from noncooperative animals can be an issue because of the unwillingness to chew on the ropes, but it is considered as a minor issue if compared to the danger caused by noncooperative animals during blood sampling. The personnel may get injured if the animals are not cooperative.

Based on Figs. 2 and 5, both pen and individual oral fluid samples show similar pattern of results. An increase in S/P values for individual oral fluid samples is coherent with an increase in S/P values for pen oral fluid samples of the same age groups and vice versa. The only difference is that pen oral fluid samples generally have higher S/P ratios if compared to that of individual oral fluid samples of the same age group. It has also been reported before in a previous paper that pen oral fluid samples generally have higher S/P values if compared to individual oral fluid samples (IDEXX 2013). This is because more antibodies are detected by the test thus causing significant difference between the value of pen and individual oral fluid samples. However, despite the higher value of S/P ratio for the pen oral fluid samples, statistical analysis revealed that there is no significant difference between pen and individual oral fluid samples at the same age group. Therefore, it is concluded that pen oral fluid samples can also be used as diagnostic tool to monitor PRRS in endemic farms because the results do not show significant difference between individual and pen oral fluid samplings, easier sample collection, ability to collect samples more frequently, and the ability to cover more animals in the farm. However, it is necessary to allow as much as animals to chew the rope during the pen oral fluid sampling so that more animals will be covered in the farm and PRRS status in the farm can be defined more accurately.

## Conclusion

The application of oral fluid as a diagnostic based offers advantages over serum for the purpose of monitoring PRRSV infection in the farm by using ELISA. Advantages include the simple and noninvasive methodology needed. This is because oral fluid collection does not require restraining and can be done easily by trained personnel because of the natural behaviors of pigs to chew anything in their surroundings. This will greatly improve the welfare of the animals and will not affect their productivity due to stress during handling. In addition, oral fluid samplings can also be done more frequently over a short time interval that facilitates ongoing disease monitoring.

Therefore, it is highly beneficial if the application of oral fluid can be used more widely in the animals for disease monitoring, not only for PRRSV but also for other diseases as well such as swine influenza, classical swine fever (CSF), and porcine circovirus type 2 (PCV2) infection which cause huge economic loss to the farmers as well.

As a conclusion, oral fluid is a powerful tool to be used for the purpose of disease monitoring, and the data collected is crucial to improve health management in the herds. Thus, the author strongly suggested the usage of oral fluids for PRRS monitoring instead of serum samples in endemic farms.
